# Case Report: Eculizumab in highly active myasthenia gravis complicated by severe infections

**DOI:** 10.3389/fimmu.2025.1596283

**Published:** 2025-07-31

**Authors:** Yufei Deng, Haocheng Luo, Chaoyue Zhang, Li Yang, Shuangshuang Wang, Xuxiang Zhang, Xianni Yan, Xiaojun Yang, Qilong Jiang

**Affiliations:** Department of Neuromuscular Diseases, The First Affiliated Hospital of Guangzhou University of Chinese Medicine, Guangzhou, China

**Keywords:** eculizumab, highly active myasthenia gravis, severe infections, case series, complement inhibition

## Abstract

Highly active myasthenia gravis refers to a subset of refractory patients who exhibit recurrent exacerbations and crises. Eculizumab, a complement C5 inhibitor, has shown its efficacy and safety for patients with anti-acetylcholine receptor antibody-positive(AchR +)refractory generalized myasthenia gravis(gMG) in the REGAIN trial. However, the efficacy and safety of eculizumab in treating MG patients with severe infections have not yet been supported by clinical evidence. This is a case series reporting four patients with highly active myasthenia gravis complicated by severe infections. Changes in Myasthenia Gravis-Activities of Daily Living (MG-ADL) and Quantitative Myasthenia Gravis (QMG) scores were recorded before and after 12 injections of eculizumab to assess efficacy. Pathogen characteristics of infections were summarized using bacterial culture and next-generation sequencing (NGS) results, presented as a heatmap to illustrate pathogen species and abundance. Inflammatory markers, including Procalcitonin (PCT), C-Reactive Protein (CRP), neutrophil count, and total lymphocyte count, were monitored to evaluate the safety. Treatment regimens were retrospectively analyzed to further assess clinical outcomes and safety. The baseline ADL data for the four patients was 22 ± 2.31 (Mean ± SD), and the baseline QMG data was 30.5 ± 8.23. After 12 injections of eculizumab treatment, the scores decreased to ADL 4.75 ± 3.3 and QMG 14 ± 3.37. During the treatment, no apparent worsening of infections related to Eculizumab was noted. Three patients successfully had their tracheostomy tubes removed, and none of the four patients experienced further myasthenic crises. Eculizumab demonstrated clinical improvement in this series, and the treatment was well-tolerated. This case series addresses the need for data on complement inhibitors in highly active myasthenia gravis patients with severe infections, provides clinical reference support for the expanded application of eculizumab.

## 1 Introduction

Myasthenia Gravis (MG) is a chronic autoimmune neuromuscular disorder characterized by the impairment of neuromuscular transmission due to autoantibodies targeting acetylcholine receptors or other proteins at the neuromuscular junction. Patients often experience fluctuating muscle weakness, which may involve the ocular, bulbar, and limb muscles (1). Myasthenic Crisis (MC) is a life-threatening manifestation of MG, characterized by progressive respiratory difficulty requiring respiratory support (2). Prior studies indicate that approximately 15-20% of gMG patients progress to MC, with various types of infections being the most common triggers, among which respiratory infections are the most frequent (2).

In 2023, German guideline introduced the concept of “highly active myasthenia gravis” (3) based on the framework of refractory MG. This guideline introduces quantitative analysis based on time and treatment history, provides clear and quantifiable diagnostic criteria for patients exhibiting a highly active, treatment-resistant disease state. Therefore, our study selected four patients meeting the 2023 German guideline criteria for analysis. The specific diagnostic criteria for highly active MG are defined as follows: (1) Moderate/high MGFA status (≥MGFAIIb) and/or at least two recurrent severe exacerbations/myasthenic crises with the need for therapeutic interventions including intravenous immunoglobulin(IVIg), plasma exchange(PE), and Immunoadsorption (IA) within 1 year after diagnosis despite adequate disease-modifying and symptomatic therapy. or (2) Persistent symptoms relevant to daily living (≥MGFAIIa) and severe exacerbation/myasthenic crisis within the last calendar year despite adequate disease modifying and symptomatic therapy. or (3) Persistent symptoms relevant to daily living, even of the mild/moderate course type (≥MGFAIIa), for more than 2 years despite adequate disease modifying and symptomatic therapy (3).

Emerging evidence supports a critical role for complement activation in the pathophysiology of MG. Complement plays an important role in innate and antibody-mediated immunity, and activation and amplification of complement results in the formation of membrane attack complexes (MACs), lipophilic proteins that damage cell membranes (4, 5). Eculizumab, a monoclonal antibody that inhibits the complement protein C5, has shown safety and efficacy in AchR(+)refractory gMG in the REGAIN trial, a phase 3, randomized, double-blind, placebo-controlled, multicenter study (6).

Patients with MG who undergo thymectomy, long-term immunosuppressive therapy, especially corticosteroid treatment, and require ventilator support are at significantly increased risk of infections due to these factors. Infection is a key factor in the exacerbation of MG and the onset of MC (7, 8). Within the MG patient population, a specific subgroup exhibits a highly active and refractory disease state, for whom ICU admission and severe multidrug-resistant infections pose significant therapeutic challenges. We provide a detailed case analysis of these four patients with the intention of providing clinical evidence supporting the efficacy and safety of eculizumab in treating highly active MG complicated by severe infections.

## 2 Methods

### 2.1 Study design and ethical approval

This study was a retrospective case series analysis of four patients with highly active MG treated with Eculizumab at our center in 2024. The study protocol was approved by the Ethics Committee of The First Affiliated Hospital of Guangzhou University of Chinese Medicine (Approval No.: K-2024-217), and written informed consent was obtained from all participants.

### 2.2 Patient selection

Inclusion Criteria: (a) Generalized MG with AChR (+). (b) Diagnosis of highly active MG according to the 2023 German guideline criteria (3). Definition of manifest myasthenic crisis:MGFA Class V: Worsening of myasthenic weakness requiring intubation or noninvasive ventilation to avoid intubation, except when these measures are employed during routine postoperative management (the use of a feeding tube without intubation places the patient in MGFA Class IVB) (9). (c) During 2024, the patient completed a total of 12 eculizumab injections at our center. (d) Concurrent infections with ≥2 bacterial pathogens or co-infections involving bacteria, viruses, or fungi. (Confirmed by microbiological culture or NGS); (e) Patients must be vaccinated against Neisseria meningitidis at least 2 weeks prior to the first dose. If urgent treatment is needed in unvaccinated patients, administer appropriate antibiotics for ≥2 weeks post-vaccination (10).

Exclusion Criteria: (a) Patients who were not vaccinated against Neisseria meningitidis and could not receive prophylactic antibiotic therapy. (b) The treatment course with 12 sequential eculizumab injections was incomplete.

### 2.3 Data collection and outcome measures

Baseline Characteristics: (a) Demographic data: Gender, age. (b) Clinical profiles: Antibody subtypes (AChR, Titin, RYR), thymic status (thymoma confirmed by surgery, Computed Tomography or Magnetic Resonance Imaging), disease duration (months), comorbidities, MGFA classification before first crisis, and ICU length of stay (days).

Intervention: Eculizumab regimen: (a) Induction phase: 900 mg weekly for 4 weeks. (b) Maintenance phase: 1200 mg every 2 weeks. (Be consistent with REGAIN trial (6).)

All four patients completed 12 injections over a cumulative treatment period of 20 weeks.

Efficacy Outcomes: (a) Primary endpoints: Changes in MG-ADL (Myasthenia Gravis Activities of Daily Living) and QMG (Quantitative Myasthenia Gravis) scores. (b) Secondary endpoints: Time to successful tracheal tube extubation and number of recurrent MC post-eculizumab initiation.

Safety Outcomes: (a) Inflammatory markers: Procalcitonin, C-Reactive Protein, absolute neutrophil count, and total lymphocyte count. (b) TRAE (Treatment-Related Adverse Event): Worsening of pre-existing infections or new infections (e.g., meningococcal disease).

### 2.4 Statistical analysis

Descriptive statistics. Statistical data were analyzed and visualized using GraphPad Prism software (version 10.4.0).

Visualization: (a) Line charts to display trends in MG-ADL and QMG scores. (b) Heatmap to illustrate pathogen species and abundance.

### 2.5 Data sources

Medical history and treatment records: Electronic medical record system of The First Affiliated Hospital of Guangzhou University of Chinese Medicine.

Laboratory data: Inflammatory markers and sputum culture results: Clinical Laboratory of the same hospital.

NGS reports: Guangzhou Daan Clinical Testing Center.

## 3 Results

### 3.1 Baseline characteristics

**Figure 1:**
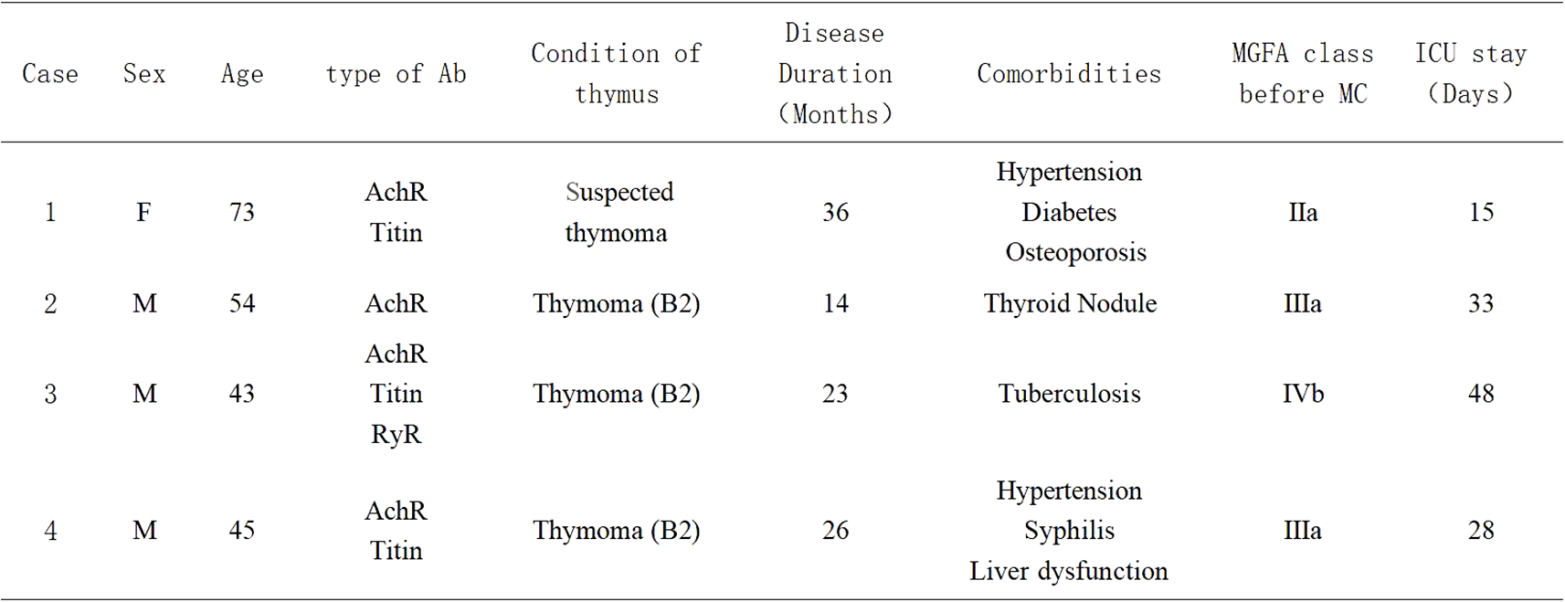
Baseline clinical features of the four myasthenic cases; Ab, antibody; MGFA class, Myasthenia Gravis Foundation of America Classification; ICU, Intensive Care Unit; MC, Myasthenic Crisis; Sex-M, Male; Sex-F, Female.

The baseline clinical features of the four patients are summarized in **Figure 1**.

### 3.2 Infection profiles

**Figure 2:**
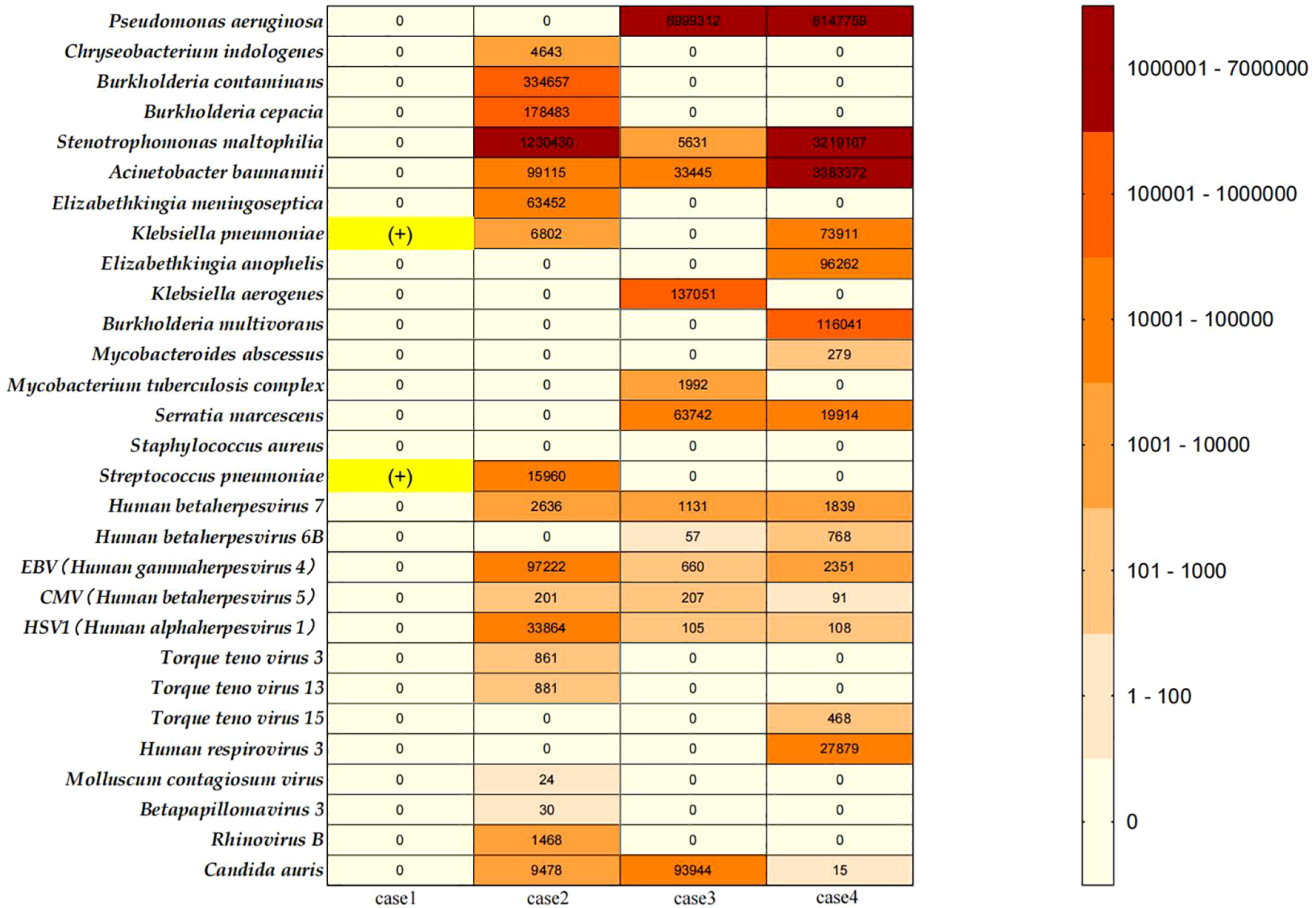
Heat map of pathogens encountered in the myasthenic patients; Case 1: Sputum culture results from the clinical laboratory of our center (The First Affiliated Hospital of Guangzhou University of Chinese Medicine) showed positive cultures for Klebsiella pneumoniae and Streptococcus pneumoniae. This patient did not undergo NGS testing. Since case 1 did not undergo NGS testing, her bacterial culture results are displayed with a yellow highlight mark (+). We collected all NGS test results performed during the treatment of Cases 2, 3, and 4 at our center. The details are as follows: Case 2: A total of 16 NGS tests were conducted from March 29, 2024, to November 8, 2024. Specimens included bronchoalveolar lavage fluid (BALF) and sputum.; Case 3: A total of 8 NGS tests were conducted from March 27, 2024, to September 15, 2024. Specimens included BALF and sputum; Case 4: A total of 7 NGS tests were conducted from January 18, 2024, to July 3, 2024. Specimens included BALF and sputum. Pathogen Analysis: In this report, we documented the bacterial, viral, and fungal pathogens detected in the NGS results of Cases 2-4, excluding suspected colonizing microorganisms and human microbiota. The left column summarizes the types of pathogens identified. For abundance comparison, we selected the highest reads count for each pathogen type from all test results for each patient and generated a heatmap.

As illustrated in **Figure 2**.

### 3.3 Clinical outcomes

**Figure 3:**
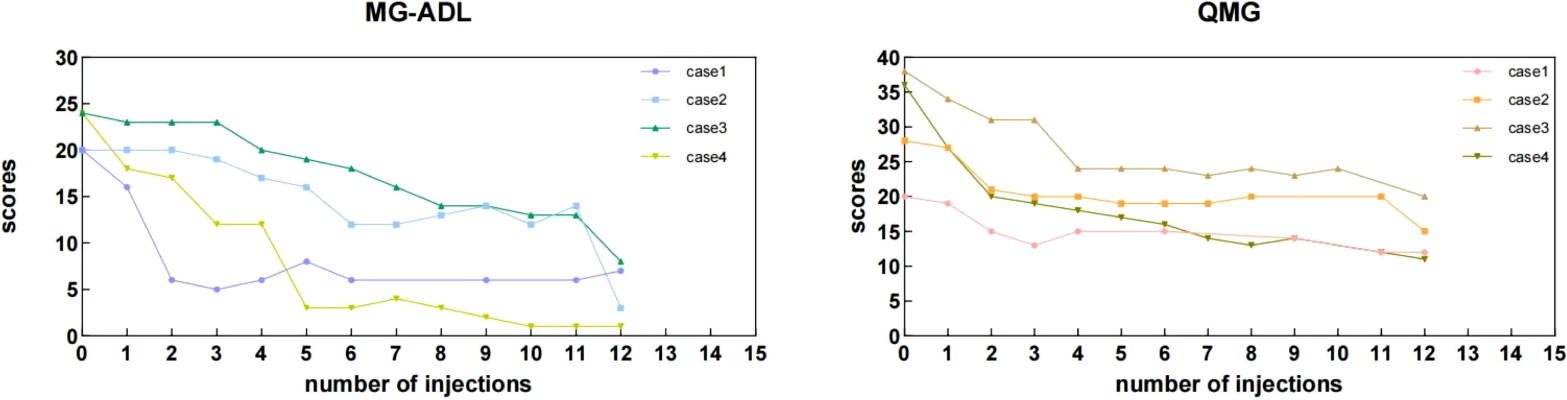
Individual MG-ADL and QMG scores of the myasthenic patients during the 20 weeks period. During the maintenance phase, some QMG and MG-ADL scores were missing at specific injection time points. This was attributed to several factors, including patients returning to their local areas after discharge, transitioning to telephone follow-ups, or insufficient willingness to complete the assessments. Additionally, some patients opted for monthly assessments after consultation, resulting in missing data at certain injection time points. The “0” represents the baseline assessment score recorded prior to the initiation of eculizumab treatment.

Efficacy: (a) Primary Endpoints: MG-ADL scores decreased from a baseline of 22 ± 2.31 to 4.75 ± 3.3 after 12 eculizumab injections. QMG scores decreased from 30.5 ± 8.23 to 14 ± 3.37. Individual patient score trajectories are shown in **Figure 3**. (b) Secondary Endpoints: Three patients achieved successful extubation. (Case 1: Prior to eculizumab initiation (post-PLEX and ICU care). Case 2: After the 11th infusion. Case 3: After the 4th infusion. Case 4: After the 5th infusion.); Crisis control: No recurrent myasthenic crises occurred during 20 weeks of eculizumab treatment.

### 3.4 Safety outcomes

(a)No meningococcal infections or TRAE occurred. (b) Two transient worsening of infection was observed during the Eculizumab treatment. Case 2: Transient low-grade fever (peak 37.6°C) after the 10th infusion, attributed to residual ventilator-associated pneumonia (VAP). Symptoms resolved with antipyretics and antibiotic adjustment; Case 4: Impending Myasthenia crisis (IMC) symptoms (fatigue, dyspnea) occurred before the 6th infusion, accompanied by elevated inflammatory markers and hypercapnia (pCO_2_ 93.6 mmHg). Non-invasive ventilation and prompt eculizumab administration in the next day prevented crisis progression. Subsequent infusions (7th, 9th, 10th) showed persistently elevated markers without clinical deterioration, requiring no intervention (**Supplementary Table 1**).

## 4 Cases description

**Figure 4:**
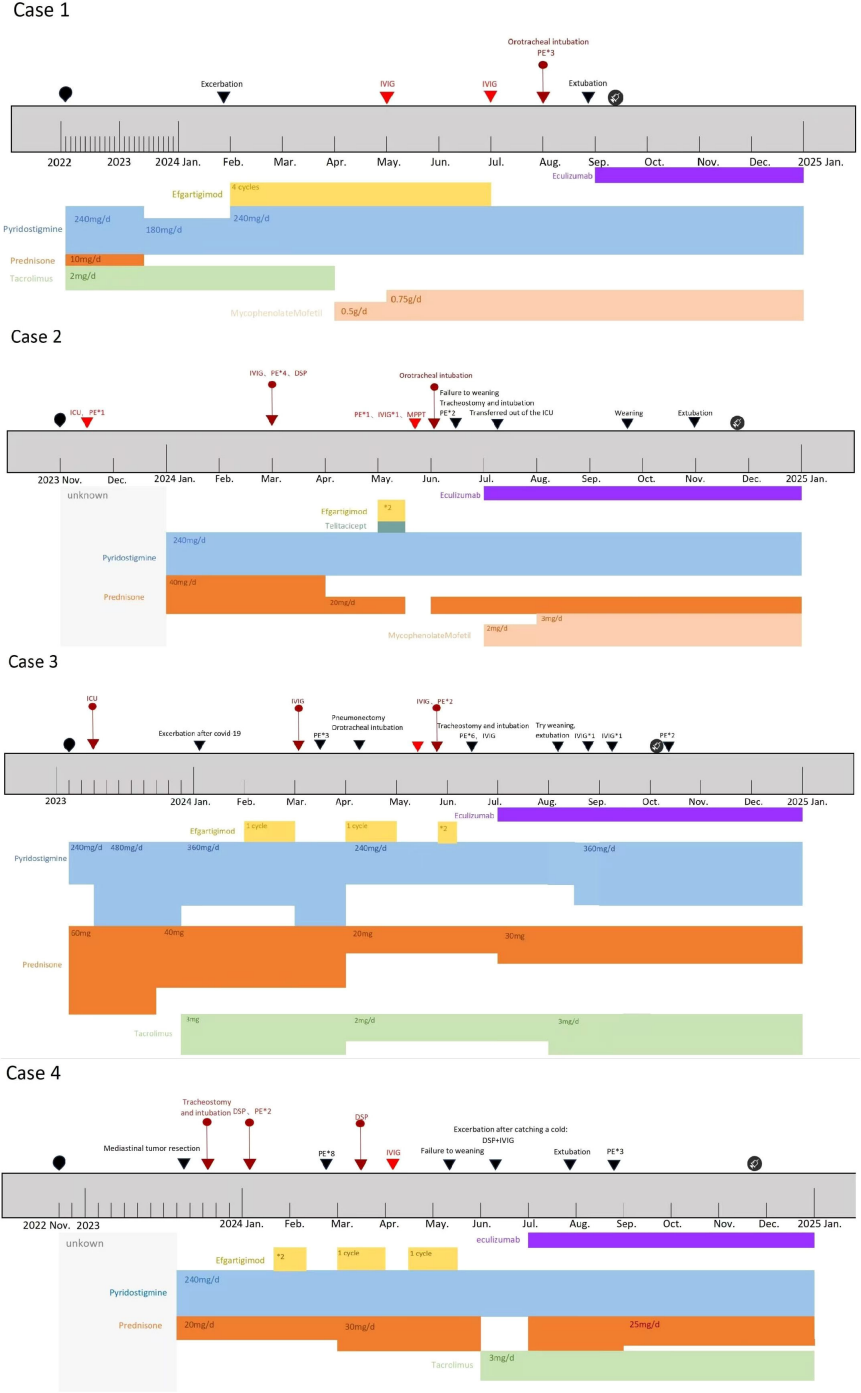
Timeline of four patients’ disease and treatment process; 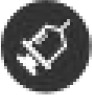: Time points of meningococcal vaccination in four Patients. The type of vaccine administered: ACYW135 meningococcal polysaccharide vaccine. 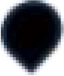:Onset time of the disease. ▼: Timing of disease progression and significant treatment. 
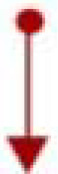
:Myasthenic Crisis (MC). 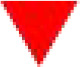:Impending Myasthenia crisis (IMC). DSP, Dexamethasone sodium phosphate pulse therapy. MPPT, Methylprednisolone pulse therapy. Efgartigimod administration: 1 cycle = 4 injections, once per week. The injection dose is calculated based on the patient’s body weight by 10 mg/kg. Unknown:For Case 2, the initial treatment details are unclear due to delayed diagnosis. For Case 4, the initial treatment details are unclear due to incomplete patient recall.

The treatment process and disease course of the 4 patients are presented in **Figure 4**.

### 4.1 Case 1: female, 73 years old, late-onset MG with suspected thymoma

This patient experienced recurrent exacerbations despite multiple treatments, including four cycles of efgartigimod and IVIg during IMCs. Advanced age and multiple chronic comorbidities were significant risk factors for her disease progression. Chest Computed Tomography (CT) revealed minimal thymic hyperplasia, but further evaluation was declined by the patient. A suspected thymoma was considered based on her clinical presentation and history. Prednisone was gradually discontinued in 2023 due to severe osteoporosis. In August 2024, she developed an MC requiring mechanical ventilation (MV). She remained in the Intensive Care Unit(ICU)for 15 days, undergoing four sessions of PE, which stabilized her vital signs and allowed successful extubation with transition to non-invasive ventilation. However, after transfer from the ICU, sputum cultures revealed infections with *Klebsiella pneumoniae* and *Streptococcus pneumoniae*. Given her highly active and refractory disease, eculizumab was initiated, leading to significant improvement in limb strength and swallowing function. After 12 injections, her MG-ADL and QMG scores decreased from 20 to 6 and 20 to 13, respectively. She achieved ventilator independence, and her infection markers stabilized without signs of exacerbation. (**Supplementary Table 1**) She continued eculizumab therapy without further crises.

### 4.2 Cases 2、3、4: male, middle-aged, thymoma-associated myasthenia gravis

These three patients with B2-type thymoma experienced severe disease courses characterized by recurrent MCs. The three patients stayed in ICU for nearly 1 month or more, and all of them exhibited difficulties in weaning from the ventilator, experiencing several failed attempts. All three patients underwent multiple bronchoalveolar lavages (BAL) with multiple next-generation sequencing (NGS), revealing complex infections with multidrug-resistant bacteria, viruses, and *Candida auris* (**Figure 2**).

These three patients underwent multiple cycles of IVIg and PE during MCs and IMCs, and had been treated with targeted biologics, such as efgartigimod (Case 2 had even tried a combination of efgartigimod and telitacicept). However, their clinical courses indicated poor responses to these therapies. Following eculizumab initiation, all three patients showed significant clinical improvement after 1–2 injections. In subsequent treatment, respiratory and limb muscle strength gradually recovered, and they were successfully extubated after the 11th, 4th, and 5th injections, respectively. and no further crises. Their MG-ADL and QMG scores also decreased and achieved Minimal Manifest Symptoms (MMS) after 12 injections of eculizumab. During the follow-up, we learned that Cases 2 and 4 currently require only a few hours of nocturnal non-invasive ventilation support, while the Case 3 has completely weaned off ventilator dependency.

### 4.3 Supplemental notes on Neisseria meningitidis vaccination

Complement inhibitors increase susceptibility to Neisseria meningitidis infections (11). Therefore, the FDA prescribing information recommends receiving meningococcal vaccination ≥2 weeks prior to initiating eculizumab therapy. If vaccination cannot be administered in advance, appropriate prophylactic antibiotics must be concurrently used (10). In these four cases, due to rapid disease progression and severe clinical status, completing meningococcal vaccination ≥2 weeks before eculizumab administration was unfeasible. However, all patients had a history of severe ongoing infections and ICU admission. Throughout their treatment at our center, antimicrobial regimens covering Neisseria species were implemented, including penicillins, third-generation cephalosporins, vancomycin and so on. Antibiotic regimens were maintained until 2 weeks post-completion of meningococcal vaccination in all four patients (see vaccination timepoints in **Figure 4**). Consequently, despite the lack of timely vaccination, no meningococcal complications occurred during eculizumab treatment in these four patients.

## 5 Patient perspective

Four patients with highly active refractory myasthenia gravis (MG), including thymoma-associated or suspected thymoma cases, were enrolled based on poor prognoses, recurrent life-threatening exacerbations requiring ICU admissions, and inadequate responses to conventional therapies. These individuals endured prolonged ventilator dependence, repeated treatment failures, and significant psychological distress, including depression and anxiety, alongside substantial financial burdens. Following 12 injections of eculizumab therapy, all achieved marked clinical stabilization without recurrent crises, transitioning to improved disease control and enhanced quality of life. Written informed consent and ethics approval were secured prior to treatment initiation.

## 6 Discussion

In this case series, all four patients exhibited high disease activity within one year. Despite the administration of standard immunosuppressive therapy (**Figure 4**), symptom control remained suboptimal. Pyridostigmine and corticosteroids showed limited efficacy, with Case 3 requiring a high dose of pyridostigmine (480 mg/day) and Cases 2, 3, and 4 unable to reduce their corticosteroid doses. Immunosuppressants, such as tacrolimus and mycophenolate mofetil, were introduced; however, their slow onset of action (12) and the rapid disease progression precluded the observation of long-term benefits. During disease exacerbations, clinical and laboratory findings indicated infections, prompting the use of IVIg (13). Unfortunately, IVIg failed to halt disease progression, and we observed that in these 4 patients, the response effect of IVIg gradually decreased as the frequency of use increased, with some even unable to complete the five-day course. They urgently required additional PE and high-dose corticosteroid pulse therapy. Besides poor response, these rescue treatments targeting MC or IMC were only effective during acute exacerbations and not enough to reduce long-term disease activity in these four patients.

Following the initiation of eculizumab therapy, significant improvements were observed. Reductions in QMG and MG-ADL scores were noted after just 1–2 injections, consistent with findings from the REGAIN trial, where MG-ADL and QMG scores began to decrease within 1 and 2 weeks, respectively (6). Notably, the REGAIN trial excluded patients with thymoma, but a real-world study have demonstrated a similar rapid onset of eculizumab’s effect in thymoma associated myasthenia gravis (TAMG) patients, typically within 2 weeks (14). In our series, the average MG-ADL score decreased by 17.25 points after 12 injections, compared to a reduction of less than 5 points in the REGAIN trial in the same efficacy endpoint. This discrepancy may be attributed to the significantly higher baseline MG-ADL scores in our series, suggesting that eculizumab may exhibit more pronounced efficacy in patients with severe baseline conditions.

Some clinical reports have already confirmed the efficacy of eculizumab in MC patients (15–17). In our report, we focused more on the role of eculizumab treatment in reducing disease activity and achieving better long-term outcomes. The sustained efficacy of eculizumab is supported by pharmacokinetic studies, which demonstrated maintained therapeutic effects throughout the 26-week treatment period (18). In our series, by the 12th injection, all four patients achieved MMS with no further crises, highlighting eculizumab’s potential as a sequential treatment option for highly active myasthenia gravis, bridging acute exacerbations to long-term maintenance. Mechanistically, eculizumab’s targeted inhibition of complement C5 offers a distinct advantage in managing highly-active patients (19).

However, in our series, the doses of corticosteroids, pyridostigmine, and immunosuppressive agents remained unchanged due to the high disease activity and thymoma complications. A cautious approach to tapering these medications was adopted, emphasizing the need for longer clinical observation.

Infection is one of the primary triggers for myasthenic crisis (2). In this series, all four patients experienced MC triggered by bacterial or viral respiratory infections, requiring MV and ICU support. VAP poses a significant challenge for these patients. VAP not only prolongs the duration of ICU hospitalization but also increases the risk of weaning difficulties (20). Controlling infection and facilitating early liberation from mechanical ventilation are key therapeutic goals. We summarized the pathogens detected in the four patients, including multidrug-resistant bacteria, viruses, and fungi (**Figure 2**). We found that these results largely align with the pathogenic characteristics of pneumonia associated with MG (7) and VAP in Asian populations (21) in previous studies, indicating that the pathogen profiles in these four patients hold significant therapeutic reference value. While a few clinical reports have explored eculizumab’s efficacy in myasthenic crisis (15–17), our study breaks new ground by investigating its efficacy and safety in highly active MG patients with severe coinfections. Particularly, Candida auris infection, known for its high mortality and refractory nature (22, 23), has garnered increasing attention in recent years (24).

Given that all four patients were diagnosed with multidrug-resistant bacterial infections during their ICU treatment, antibiotic therapy was initiated immediately and continued. The selection of antibiotics was based on the results of antimicrobial susceptibility testing and the NGS results of the four patients. We initiated eculizumab treatment while simultaneously monitoring changes in inflammatory markers and clinical manifestations. During the treatment in Case 2 and Case 4, two transient worsening of infection occurred. However, both were deemed unrelated to eculizumab, although complement inhibition elevates the risk of infections by encapsulated bacteria, which may leads to exacerbation (25). The fever in Case 2 emerged following the 10th infusion. Crucially, serial NGS results conducted prior to Eculizumab treatment had already identified the presence of encapsulated bacterial infections. No new encapsulated bacterial infection was detected. Secondly, Infections with encapsulated bacteria facilitated by complement inhibitors are severe and difficult to control. But the manifestation in Case 2 was only a low-grade fever without the elevation of infection markers (**Supplementary Table 1**), and the condition resolved quickly. Case 4 experienced IMC prior to the 6th eculizumab infusion. Analysis of inflammatory markers (**Supplementary Table 1**) suggested this IMC was highly associated with a worsening of infection. Pharmacologically, eculizumab precisely targets complement C5. Existing research indicates that C5 deficiency is associated with an elevated risk of Neisseria meningitidis (meningococcus) infection, but not conclusively with other encapsulated bacteria. Other infections are more strongly associated with C3b inhibition (26). Analysis of infection profiles (**Figure 2**) revealed infections with various encapsulated bacteria. However, no Neisseria meningitidis infection was detected. Therefore, this infection exacerbation showed no link to eculizumab injection. Conversely, his condition improved following the 6th eculizumab infusion the next day, and the episode did not progress to MC. In contrast, the inflammatory markers and infection status in case 1 and case 3 remained stable throughout the treatment period. (**Supplementary Table 1**) Additionally, although none of the four patients had the opportunity to receive meningococcal vaccination prior to treatment initiation, none of them developed meningitis infection. This may be attributed to the use of antibiotic therapy throughout the entire treatment.

As the patients’ overall muscle strength improved and pulmonary ventilation function recovered, all four patients successfully had their tracheal tubes removed and were liberated from mechanical ventilation, further reducing the risk of infection. These results indicate that eculizumab treatment is safe and well-tolerated in MG patients with severe infections.

## 7 Conclusion

In patients with highly active myasthenia gravis complicated by multidrug-resistant bacterial, viral, and fungal infections, eculizumab demonstrated safety and efficacy. All four patients received 12 doses of eculizumab, resulting in significant clinical improvement, and no further crises, alongside reductions in MG-ADL and QMG scores. Complement inhibitors show promise for managing highly active myasthenia gravis with severe infections, warranting further validation through larger-scale studies.

This case series has several limitations. The small sample size and short follow-up duration restrict the generalizability of our findings. Additionally, the concurrent use of other immunosuppressants, biologic agents, and corticosteroids was not excluded, which may have introduced confounding factors affecting the observed outcomes. The clinical characterization of this study was limited by incomplete assessment scales and institutional operational constraints, which precluded comprehensive documentation of patients’ psychosocial, family histories, and genetic profiles. Therefore, the results of this report should be interpreted with caution. Larger-scale, randomized controlled trials are needed to further validate the efficacy and safety of eculizumab in the treatment of refractory myasthenia gravis, particularly in patients with thymoma-associated disease or complex clinical presentations.

## Data Availability

The original contributions presented in the study are included in the article/**Supplementary Material**. Further inquiries can be directed to the corresponding author.

## References

[1] Gilhus NE Tzartos S Evoli A Palace J Burns TM Verschuuren JJGM. Myasthenia gravis. Nat Rev Dis Primers. (2019) 5:30. doi: 10.1038/s41572-019-0079-y, PMID: 31048702

[2] Claytor B Cho S Li Y. Myasthenic crisis. Muscle Nerve. (2023) 68:8–19. doi: 10.1002/mus.27832, PMID: 37114503

[3] Wiendl H Abicht A Chan A Della Marina A Hagenacker T Hekmat K. Guideline for the management of myasthenic syndromes. Ther Adv Neurol Disord. (2023) 16:17562864231213240. doi: 10.1177/17562864231213240, PMID: 38152089 PMC10752078

[4] Lee JD Woodruff TM. The emerging role of complement in neuromuscular disorders. Semin Immunopathol. (2021) 43:817–28. doi: 10.1007/s00281-021-00895-4, PMID: 34705082

[5] Howard JF. Myasthenia gravis: the role of complement at the neuromuscular junction. Ann New York Acad Sci. (2018) 1412:113–28. doi: 10.1111/nyas.13522, PMID: 29266249

[6] Howard JF Utsugisawa K Benatar M Murai H Barohn RJ Illa I. Safety and efficacy of eculizumab in anti-acetylcholine receptor antibody-positive refractory generalised myasthenia gravis (REGAIN): a phase 3, randomised, double-blind, placebo-controlled, multicentre study. Lancet Neurol. (2017) 16:976–86. doi: 10.1016/S1474-4422(17)30369-1, PMID: 29066163

[7] Su M Jin S Jiao K Yan C Song J Xi J. Pneumonia in myasthenia gravis: Microbial etiology and clinical management. Front Cell Infect Microbiol. (2022) 12:1016728. doi: 10.3389/fcimb.2022.1016728, PMID: 36569203 PMC9780595

[8] Gilhus NE Romi F Hong Y Skeie GO. Myasthenia gravis and infectious disease. J Neurol. (2018) 265:1251–8. doi: 10.1007/s00415-018-8751-9, PMID: 29372387

[9] Sanders DB Wolfe GI Benatar M Evoli A Gilhus NE Illa I. International consensus guidance for management of myasthenia gravis: Executive summary. Neurology. (2016) 87:419–25. doi: 10.1212/WNL.0000000000002790, PMID: 27358333 PMC4977114

[10] Drugs@FDA: FDA-approved drugs. Available online at: https://www.accessdata.fda.gov/scripts/cder/daf/index.cfm?event=overview.process&ApplNo=125166 (Accessed July 2, 2025).

[11] McNamara LA Topaz N Wang X Hariri S Fox L MacNeil JR. High risk for invasive meningococcal disease among patients receiving eculizumab (Soliris) despite receipt of meningococcal vaccine. MMWR Morb Mortal Wkly Rep. (2017) 66:734–7. doi: 10.15585/mmwr.mm6627e1, PMID: 28704351 PMC5687588

[12] Menon D Barnett C Bril V. Novel treatments in myasthenia gravis. Front Neurol. (2020) 11:538. doi: 10.3389/fneur.2020.00538, PMID: 32714266 PMC7344308

[13] Gajdos P Chevret S Clair B Tranchant C Chastang C. Clinical trial of plasma exchange and high-dose intravenous immunoglobulin in myasthenia gravis. Myasthenia gravis Clin study Group Ann Neurol. (1997) 41:789–96. doi: 10.1002/ana.410410615, PMID: 9189040

[14] Jin L He D Zeng Q Tan S Shi J Liu Y. Eculizumab in thymoma-associated myasthenia gravis: A real-world cohort study. Ther Adv Neurol Disord. (2024) 17:17562864241309431. doi: 10.1177/17562864241309431, PMID: 39735403 PMC11672488

[15] Song J Huan X Chen Y Luo Y Zhong H Wang Y. The safety and efficacy profile of eculizumab in myasthenic crisis: a prospective small case series. Ther Adv Neurol Disord. (2024) 17:17562864241261602. doi: 10.1177/17562864241261602, PMID: 39072008 PMC11282533

[16] Vinciguerra C Bevilacqua L Toriello A Iovino A Piscosquito G Calicchio G. Starting eculizumab as rescue therapy in refractory myasthenic crisis. Neurol Sci. (2023) 44:3707–9. doi: 10.1007/s10072-023-06900-y, PMID: 37306795

[17] Hofstadt-van Oy U Stankovic S Kelbel C Oswald D Larrosa-Lombardi S Barchfeld T. Complement inhibition initiated recovery of a severe myasthenic crisis with COVID-19. J Neurol. (2021) 268:3125–8. doi: 10.1007/s00415-021-10428-6, PMID: 33537898 PMC7857861

[18] Monteleone JPR Gao X Kleijn HJ Bellanti F Pelto R. Eculizumab pharmacokinetics and pharmacodynamics in patients with generalized myasthenia gravis. Front Neurol. (2021) 12:696385. doi: 10.3389/fneur.2021.696385, PMID: 34795626 PMC8594444

[19] Ozawa Y Uzawa A Onishi Y Yasuda M Kojima Y Kuwabara S. Activation of the classical complement pathway in myasthenia gravis with acetylcholine receptor antibodies. Muscle Nerve. (2023) 68:798–804. doi: 10.1002/mus.27973, PMID: 37705312

[20] Safdar N Dezfulian C Collard HR Saint S. Clinical and economic consequences of ventilator-associated pneumonia: a systematic review. Crit Care Med. (2005) 33:2184–93. doi: 10.1097/01.CCM.0000181731.53912.D9, PMID: 16215368

[21] Bonell A Azarrafiy R Huong VTL Viet TL Phu VD Dat VQ. A systematic review and meta-analysis of ventilator-associated pneumonia in adults in asia: an analysis of national income level on incidence and etiology. Clin Infect Dis. (2019) 68:511–8. doi: 10.1093/cid/ciy543, PMID: 29982303 PMC6336913

[22] Jacobs SE Jacobs JL Dennis EK Taimur S Rana M Patel D. Candida auris pan-drug-resistant to four classes of antifungal agents. Antimicrob Agents Chemother. (2022) 66:e0005322. doi: 10.1128/aac.00053-22, PMID: 35770999 PMC9295560

[23] Pandya N Cag Y Pandak N Pekok AU Poojary A Ayoade F. International multicentre study of candida auris infections. J Fungi (Basel). (2021) 7:878. doi: 10.3390/jof7100878, PMID: 34682299 PMC8539607

[24] Chowdhary A Sharma C Meis JF. Candida auris: A rapidly emerging cause of hospital-acquired multidrug-resistant fungal infections globally. PLoS Pathog. (2017) 13:e1006290. doi: 10.1371/journal.ppat.1006290, PMID: 28542486 PMC5436850

[25] Heesterbeek DAC Angelier ML Harrison RA Rooijakkers SHM. Complement and bacterial infections: From molecular mechanisms to therapeutic applications. J Innate Immun. (2018) 10:455–64. doi: 10.1159/000491439, PMID: 30149378 PMC6784045

[26] Skattum L van Deuren M van der Poll T Truedsson L. Complement deficiency states and associated infections. Mol Immunol. (2011) 48:1643–55. doi: 10.1016/j.molimm.2011.05.001, PMID: 21624663

